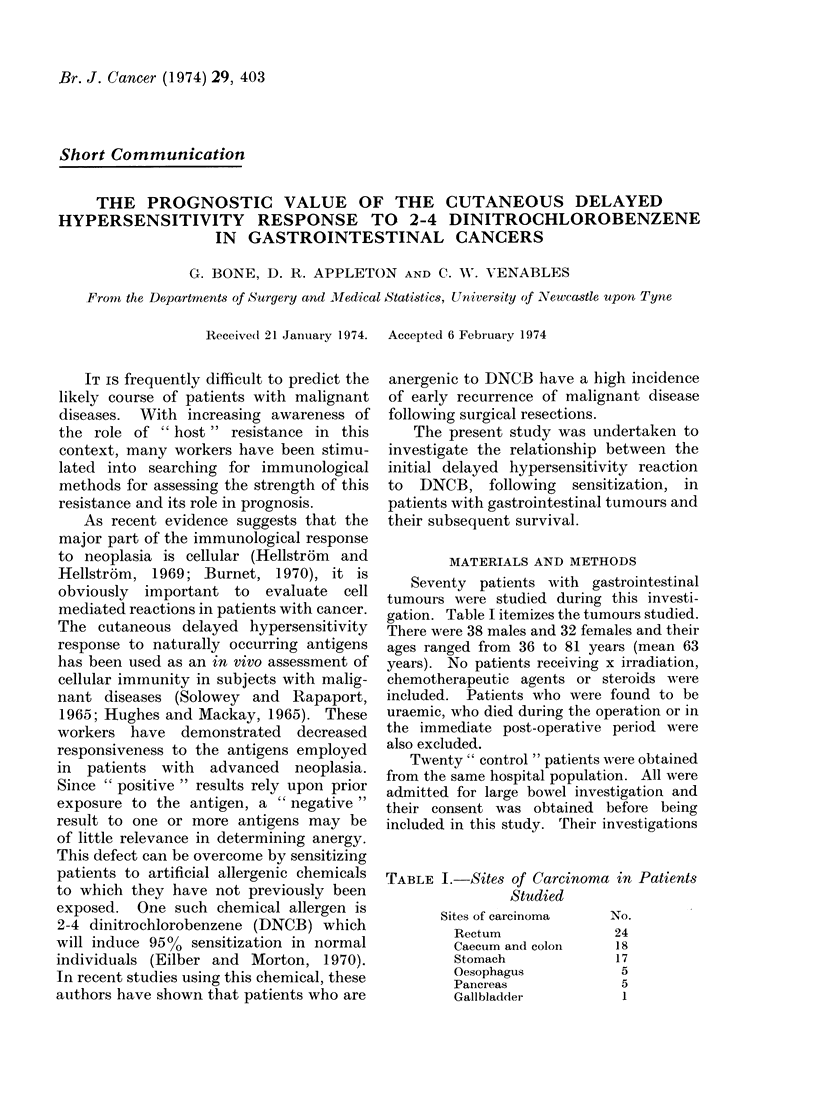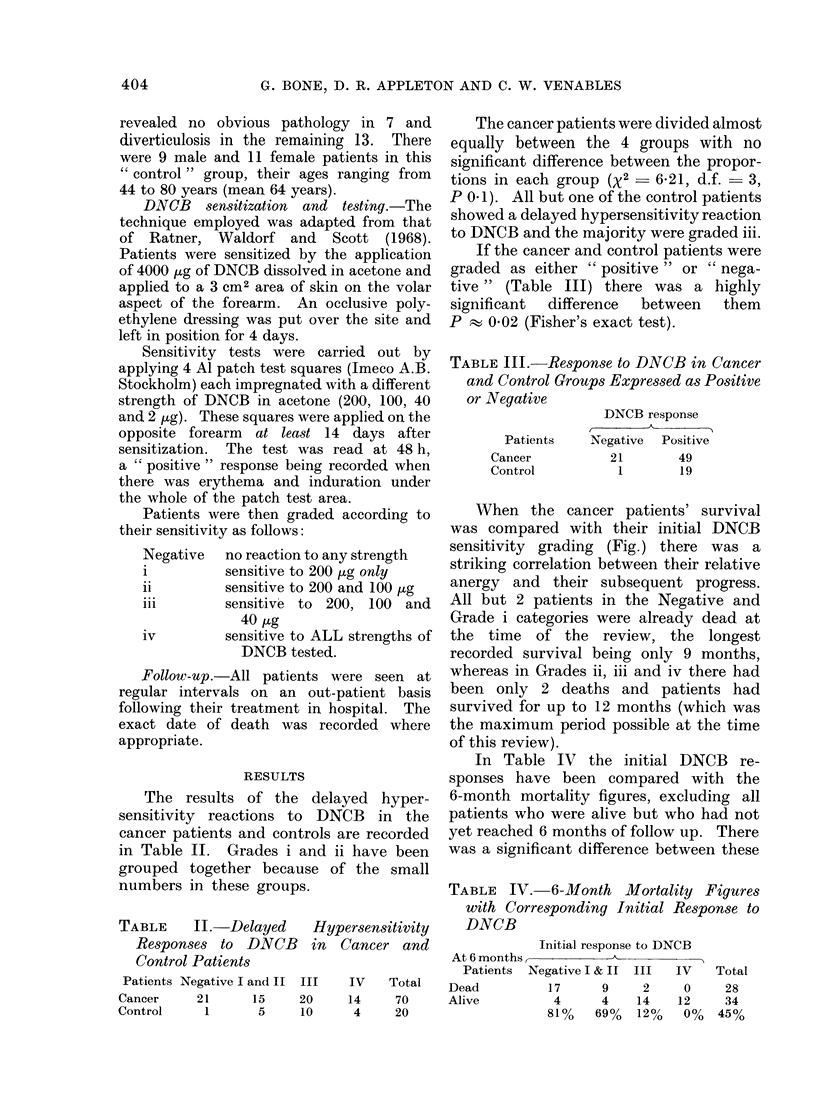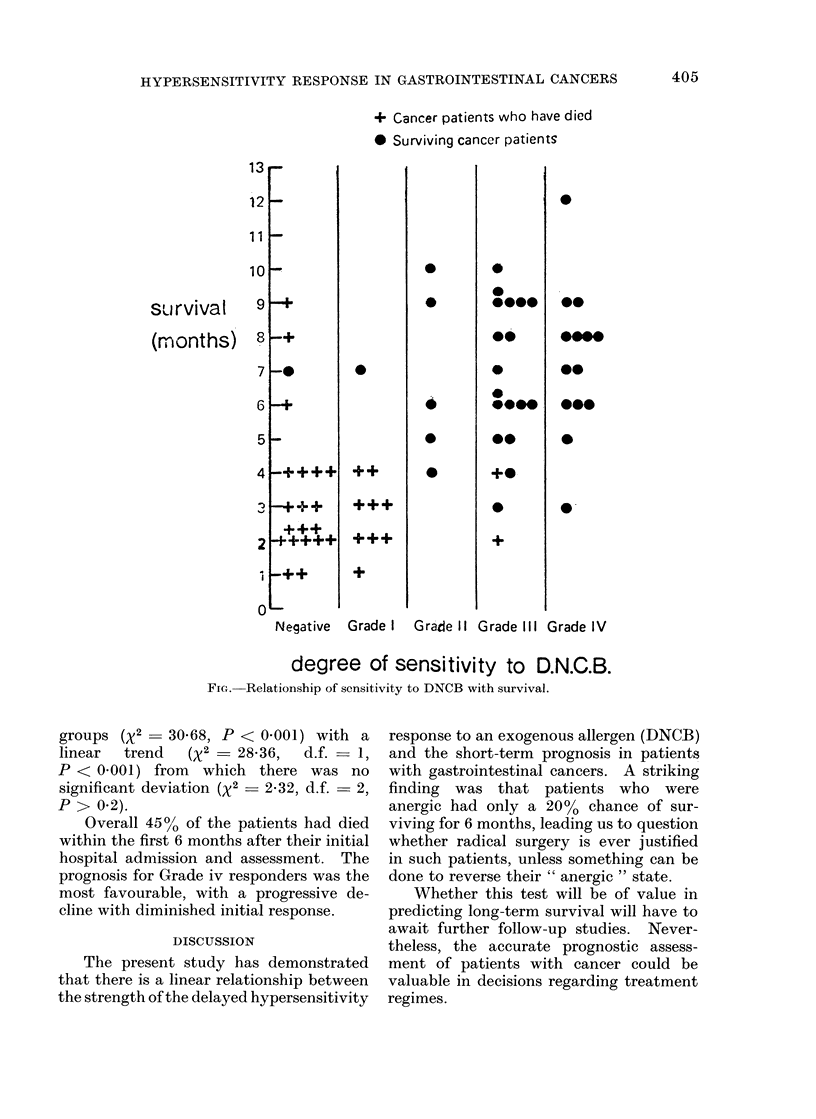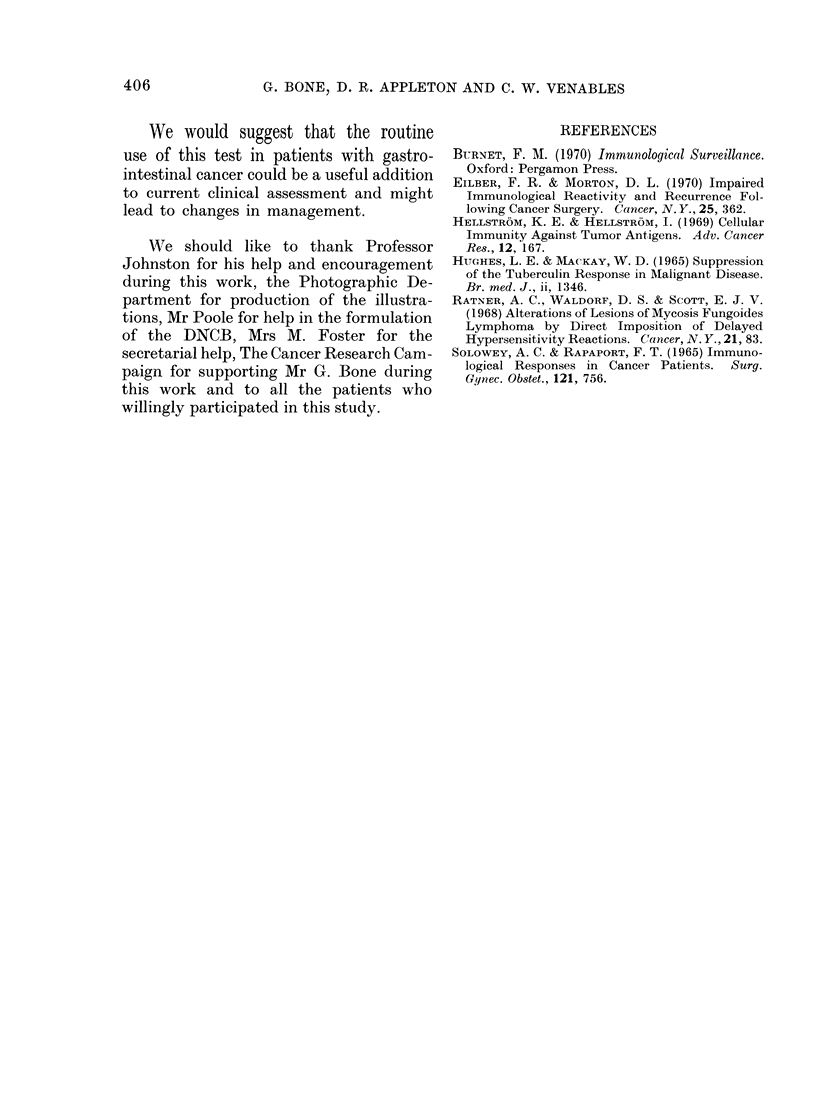# The prognostic value of the cutaneous delayed hypersensitivity response to 2-4 dinitrochlorobenzene in gastrointestinal cancers.

**DOI:** 10.1038/bjc.1974.88

**Published:** 1974-05

**Authors:** G. Bone, D. R. Appleton, C. W. Venables


*Br. J. Cancer* (1974) **29**, 403

***Short Communication***

### THE PROGNOSTIC VALUE OF THE CUTANEOUS DELAYED HYPERSENSITIVITY RESPONSE TO 2-4 DINITROCHLOROBENZENE IN GASTROINTESTINAL CANCERS

G. BONE, D. R. APPLETON AND C. W. VENABLES

*From the Departments of Surgery and Medical Statistics, University of Newcastle upon Tyne*

Received 21 January 1974. Accepted 6 February 1974

It is frequently difficult to predict the likely course of patients with malignant diseases. With increasing awareness of the role of " host " resistance in this context, many workers have been stimulated into searching for immunological methods for assessing the strength of this resistance and its role in prognosis.

As recent evidence suggests that the major part of the immunological response to neoplasia is cellular (Hellström and Hellström, 1969; Burnet, 1970), it is obviously important to evaluate cell mediated reactions in patients with cancer. The cutaneous delayed hypersensitivity response to naturally occurring antigens has been used as an *in vivo* assessment of cellular immunity in subjects with malignant diseases (Solowey and Rapaport, 1965; Hughes and Mackay, 1965). These workers have demonstrated decreased responsiveness to the antigens employed in patients with advanced neoplasia. Since " positive " results rely upon prior exposure to the antigen, a " negative " result to one or more antigens may be of little relevance in determining anergy. This defect can be overcome by sensitizing patients to artificial allergenic chemicals to which they have not previously been exposed. One such chemical allergen is 2-4 dinitrochlorobenzene (DNCB) which will induce 95% sensitization in normal individuals (Eilber and Morton, 1970). In recent studies using this chemical, these authors have shown that patients who are anergenic to DNCB have a high incidence of early recurrence of malignant disease following surgical resections.

The present study was undertaken to investigate the relationship between the initial delayed hypersensitivity reaction to DNCB, following sensitization, in patients with gastrointestinal tumours and their subsequent survival.

### MATERIALS AND METHODS

Seventy patients with gastrointestinal tumours were studied during this investigation. Table I itemizes the tumours studied. There were 38 males and 32 females and their ages ranged from 36 to 81 years (mean 63 years). No patients receiving x irradiation, chemotherapeutic agents or steroids were included. Patients who were found to be uraemic, who died during the operation or in the immediate post-operative period were also excluded.

Twenty " control " patients were obtained from the same hospital population. All were admitted for large bowel investigation and their consent was obtained before being included in this study. Their investigations

TABLE I.—*Sites of Carcinoma in Patients Studied*

| Sites of carcinoma | No. |
|---|---|
| Rectum | 24 |
| Caecum and colon | 18 |
| Stomach | 17 |
| Oesophagus | 5 |
| Pancreas | 5 |
| Gallbladder | 1 |

revealed no obvious pathology in 7 and diverticulosis in the remaining 13. There were 9 male and 11 female patients in this "control" group, their ages ranging from 44 to 80 years (mean 64 years).

*DNCB sensitization and testing.*—The technique employed was adapted from that of Ratner, Waldorf and Scott (1968). Patients were sensitized by the application of 4000 $\mu$g of DNCB dissolved in acetone and applied to a 3 $cm^2$ area of skin on the volar aspect of the forearm. An occlusive polyethylene dressing was put over the site and left in position for 4 days.

Sensitivity tests were carried out by applying 4 Al patch test squares (Imeco A.B. Stockholm) each impregnated with a different strength of DNCB in acetone (200, 100, 40 and 2 $\mu$g). These squares were applied on the opposite forearm *at least* 14 days after sensitization. The test was read at 48 h, a "positive" response being recorded when there was erythema and induration under the whole of the patch test area.

Patients were then graded according to their sensitivity as follows:

| | |
|---|---|
| Negative | no reaction to any strength |
| i | sensitive to 200 $\mu$g *only* |
| ii | sensitive to 200 and 100 $\mu$g |
| iii | sensitive to 200, 100 and 40 $\mu$g |
| iv | sensitive to ALL strengths of DNCB tested. |

*Follow-up.*—All patients were seen at regular intervals on an out-patient basis following their treatment in hospital. The exact date of death was recorded where appropriate.

### RESULTS

The results of the delayed hypersensitivity reactions to DNCB in the cancer patients and controls are recorded in Table II. Grades i and ii have been grouped together because of the small numbers in these groups.

TABLE II.—*Delayed Hypersensitivity Responses to DNCB in Cancer and Control Patients*

| Patients | Negative | I and II | III | IV | Total |
|---|---|---|---|---|---|
| Cancer | 21 | 15 | 20 | 14 | 70 |
| Control | 1 | 5 | 10 | 4 | 20 |

The cancer patients were divided almost equally between the 4 groups with no significant difference between the proportions in each group ($\chi^2 = 6{\cdot}21$, d.f. $= 3$, $P$ 0·1). All but one of the control patients showed a delayed hypersensitivity reaction to DNCB and the majority were graded iii.

If the cancer and control patients were graded as either "positive" or "negative" (Table III) there was a highly significant difference between them $P \approx 0{\cdot}02$ (Fisher's exact test).

TABLE III.—*Response to DNCB in Cancer and Control Groups Expressed as Positive or Negative*

| | DNCB response | |
|---|---|---|
| Patients | Negative | Positive |
| Cancer | 21 | 49 |
| Control | 1 | 19 |

When the cancer patients' survival was compared with their initial DNCB sensitivity grading (Fig.) there was a striking correlation between their relative anergy and their subsequent progress. All but 2 patients in the Negative and Grade i categories were already dead at the time of the review, the longest recorded survival being only 9 months, whereas in Grades ii, iii and iv there had been only 2 deaths and patients had survived for up to 12 months (which was the maximum period possible at the time of this review).

In Table IV the initial DNCB responses have been compared with the 6-month mortality figures, excluding all patients who were alive but who had not yet reached 6 months of follow up. There was a significant difference between these

TABLE IV.—6-*Month Mortality Figures with Corresponding Initial Response to DNCB*

| At 6 months | Initial response to DNCB | | | | |
|---|---|---|---|---|---|
| Patients | Negative | I & II | III | IV | Total |
| Dead | 17 | 9 | 2 | 0 | 28 |
| Alive | 4 | 4 | 14 | 12 | 34 |
| | 81% | 69% | 12% | 0% | 45% |

FIG.—Relationship of sensitivity to DNCB with survival.

groups ($\chi^2 = 30{\cdot}68$, $P < 0{\cdot}001$) with a linear trend ($\chi^2 = 28{\cdot}36$, d.f. $= 1$, $P < 0{\cdot}001$) from which there was no significant deviation ($\chi^2 = 2{\cdot}32$, d.f. $= 2$, $P > 0{\cdot}2$).

Overall 45% of the patients had died within the first 6 months after their initial hospital admission and assessment. The prognosis for Grade IV responders was the most favourable, with a progressive decline with diminished initial response.

### DISCUSSION

The present study has demonstrated that there is a linear relationship between the strength of the delayed hypersensitivity response to an exogenous allergen (DNCB) and the short-term prognosis in patients with gastrointestinal cancers. A striking finding was that patients who were anergic had only a 20% chance of surviving for 6 months, leading us to question whether radical surgery is ever justified in such patients, unless something can be done to reverse their " anergic " state.

Whether this test will be of value in predicting long-term survival will have to await further follow-up studies. Nevertheless, the accurate prognostic assessment of patients with cancer could be valuable in decisions regarding treatment regimes.

We would suggest that the routine use of this test in patients with gastrointestinal cancer could be a useful addition to current clinical assessment and might lead to changes in management.

We should like to thank Professor Johnston for his help and encouragement during this work, the Photographic Department for production of the illustrations, Mr Poole for help in the formulation of the DNCB, Mrs M. Foster for the secretarial help, The Cancer Research Campaign for supporting Mr G. Bone during this work and to all the patients who willingly participated in this study.

### REFERENCES

BURNET, F. M. (1970) *Immunological Surveillance.* Oxford: Pergamon Press.

EILBER, F. R. & MORTON, D. L. (1970) Impaired Immunological Reactivity and Recurrence Following Cancer Surgery. *Cancer, N.Y.*, **25**, 362.

HELLSTRÖM, K. E. & HELLSTRÖM, I. (1969) Cellular Immunity Against Tumor Antigens. *Adv. Cancer Res.*, **12**, 167.

HUGHES, L. E. & MACKAY, W. D. (1965) Suppression of the Tuberculin Response in Malignant Disease. *Br. med. J.*, ii, 1346.

RATNER, A. C., WALDORF, D. S. & SCOTT, E. J. V. (1968) Alterations of Lesions of Mycosis Fungoides Lymphoma by Direct Imposition of Delayed Hypersensitivity Reactions. *Cancer, N.Y.*, **21**, 83.

SOLOWEY, A. C. & RAPAPORT, F. T. (1965) Immunological Responses in Cancer Patients. *Surg. Gynec. Obstet.*, **121**, 756.